# Impact of Muscle Mass as a Prognostic Factor for Failed Waiting Time Prior to Heart Transplantation

**DOI:** 10.3389/fcvm.2021.731293

**Published:** 2021-10-18

**Authors:** Luise Roehrich, Simon H. Suendermann, Isabell Anna Just, Laurenz Kopp Fernandes, Jessica Schnettler, Sebastian Kelle, Natalia Solowjowa, Julia Stein, Manfred Hummel, Jan Knierim, Evgenij Potapov, Christoph Knosalla, Volkmar Falk, Felix Schoenrath

**Affiliations:** ^1^Department of Cardiothoracic and Vascular Surgery, German Heart Center Berlin, Berlin, Germany; ^2^German Centre for Cardiovascular Research (DZHK), Partner Site Berlin, Berlin, Germany; ^3^German Heart Foundation, Frankfurt am Main, Germany; ^4^Department of Cardiovascular Surgery, Charité-Universitätsmedizin Berlin, Corporate Member of Freie Universität Berlin and Humboldt-Universität zu Berlin, Berlin, Germany; ^5^Department of Internal Medicine, Cardiology, German Heart Center Berlin, Charité–Universitätsmedizin Berlin, Berlin, Germany; ^6^Paulinen Hospital, Berlin, Germany; ^7^Department of Health Sciences and Technology, ETH Zürich, Zurich, Switzerland

**Keywords:** waiting list mortality, heart transplantation, ventricular assist device, sarcopenia, frailty

## Abstract

**Objectives:** Clinical deterioration during the waiting time impairs the prognosis of patients listed for heart transplantation. Reduced muscle mass increases the risk for mortality after cardiac surgery, but its impact on resilience against deterioration during the waiting time remains unclear.

**Methods:** We retrospectively analyzed data from 93 patients without a VAD who were listed in Eurotransplant status “high urgent (HU)” for heart transplantation between January 2015 and October 2020. The axial muscle area of the erector spinae muscles at the level of thoracic vertebra 12 indexed to body surface area (TMESA/BSA) measured in the preoperative thoracic computed tomography scan was used to measure muscle mass.

**Results:** Forty patients (43%) underwent emergency VAD implantation during the waiting time and four patients (4%) died during the waiting time. The risk of emergency VAD implantation/death during the waiting time decreased by 10% for every cm^2^/m^2^ increase in muscle area [OR 0.901 (95% CI: 0.808–0.996); *p* = 0.049]. After adjusting for gender [OR 0.318 (95% CI: 0.087–1.073); *p* = 0.072], mean pulmonary artery pressure [OR 1.061 (95% CI: 0.999–1.131); *p* = 0.060], C-reactive protein [OR 1.352 (95% CI: 0.986–2.027); *p* = 0.096], and hemoglobin [OR 0.862 (95% CI: 0.618–1.177); *p* = 0.360], TMESA/BSA [OR 0.815 (95% CI: 0.698–0.936); *p* = 0.006] remained an independent risk factor for emergency VAD implantation/death during the HU waiting time.

**Conclusion:** Muscle area of the erector spinae muscle appears to be a potential, easily identifiable risk factor for emergency VAD implantation or death in patients on the HU waiting list for heart transplantation. Identifying patients at risk could help optimize the outcome and the timing of VAD support.

## Introduction

Cardiac transplantation remains the gold standard for end-stage heart failure, but donor organ shortage causes long waiting times even for critically ill patients in the Eurotransplant region (1, 2). In 2019, 956 patients were removed from the Eurotransplant waiting list, 665 patients underwent transplantation (3), and 244 were delisted due to death, being unfit for transplantation or other reasons (3); 159 patients died during the waiting time, and 39 patients were deemed ineligible for transplantation (3).

Several risk factors for waiting list mortality were identified, including impaired renal function and low serum albumin (4). Frailty, the reduced resilience against stressors (5), and sarcopenia, reduced muscle mass and function (6), appear to be relevant risk factors for waiting list mortality in patients undergoing kidney (7, 8), lung (9), or liver transplantation (10, 11). To the best of our knowledge, this is the first study investigating the impact of muscle mass on the prognosis during the waiting time of adult patients with a “high urgent (HU)” Eurotransplant status for heart transplantation (comparable to UNOS status 3) (12, 13).

## Materials and Methods

### Study Design

**Figure 1:**
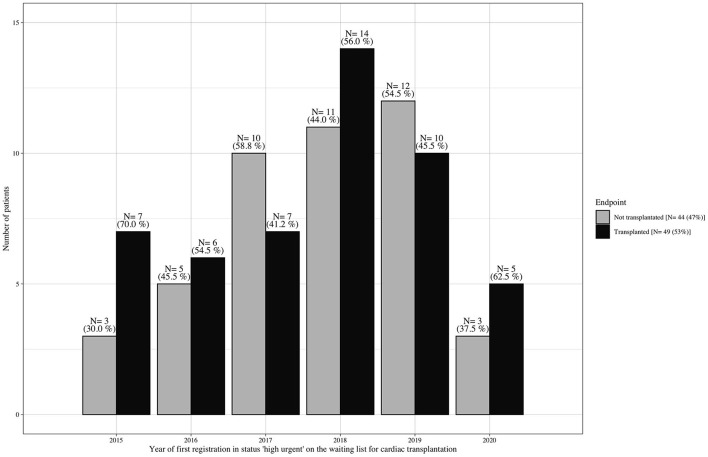
Waiting list outcome at the German Heart Center Berlin (1/2015–10/2020). Number of patients per year who were listed without mechanical circulatory support in Eurotransplant status “high urgent” for cardiac transplantation and were consecutively transplanted vs. those who did not reach transplantation due to cardiac/clinical deterioration during the waiting time, resulting in death or emergency ventricular assist device implantation.

**Figure 2:**
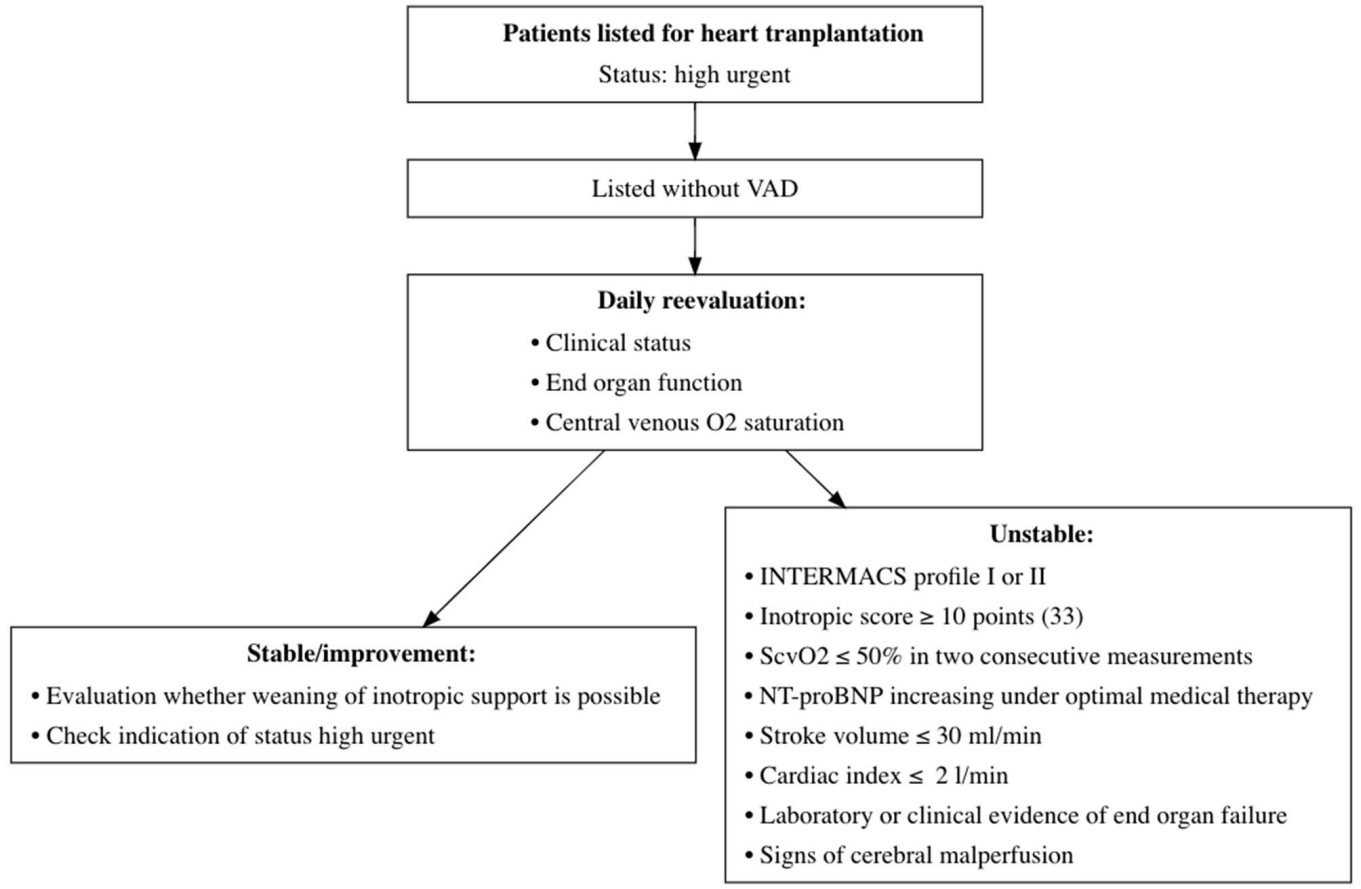
Monitoring structure during waiting time for heart transplantation at the German Heart Center Berlin. Monitoring structure for daily evaluation of patients awaiting heart transplantation in HU status. VAD, ventricular assist device; O_2_, oxygen; INTERMACS, Interagency Registry for Mechanically Assisted Circulatory Support; ScvO_2_, central venous oxygen saturation; NT-proBNP, N-Terminal Pro-B-Type Natriuretic Peptide; HU, high urgent.

From January 2015 to October 2020, 161 adult patients were granted the HU status for heart transplantation at the German Heart Center Berlin; 68 (42%) patients were already supported with a ventricular assist device (VAD) at the time of the first request for HU listing and were excluded from further analysis. Pediatric patients <16 years, patients listed for combined organ transplantation, and patients who were delisted due to stabilization did not qualify for this study. Of the remaining 93 patients, 40 (43%) underwent emergency VAD implantation and 4 (4%) died during the HU waiting time (Figure 1); consequently, 49 (53%) patients reached transplantation and 44 (47%), the outcome of interest. Baseline characteristics were collected at the time of first listing in the HU status. Organs were allocated in accordance with the Eurotransplant guidelines (14). The time of emergency VAD implantation was selected according to an intrahospital standard operating procedure of daily clinical reevaluation (Figure 2).

This study was approved by the Ethics Committee of Charité–Universitätsmedizin Berlin (EA2/236/17).

### Imaging

**Figure 3:**
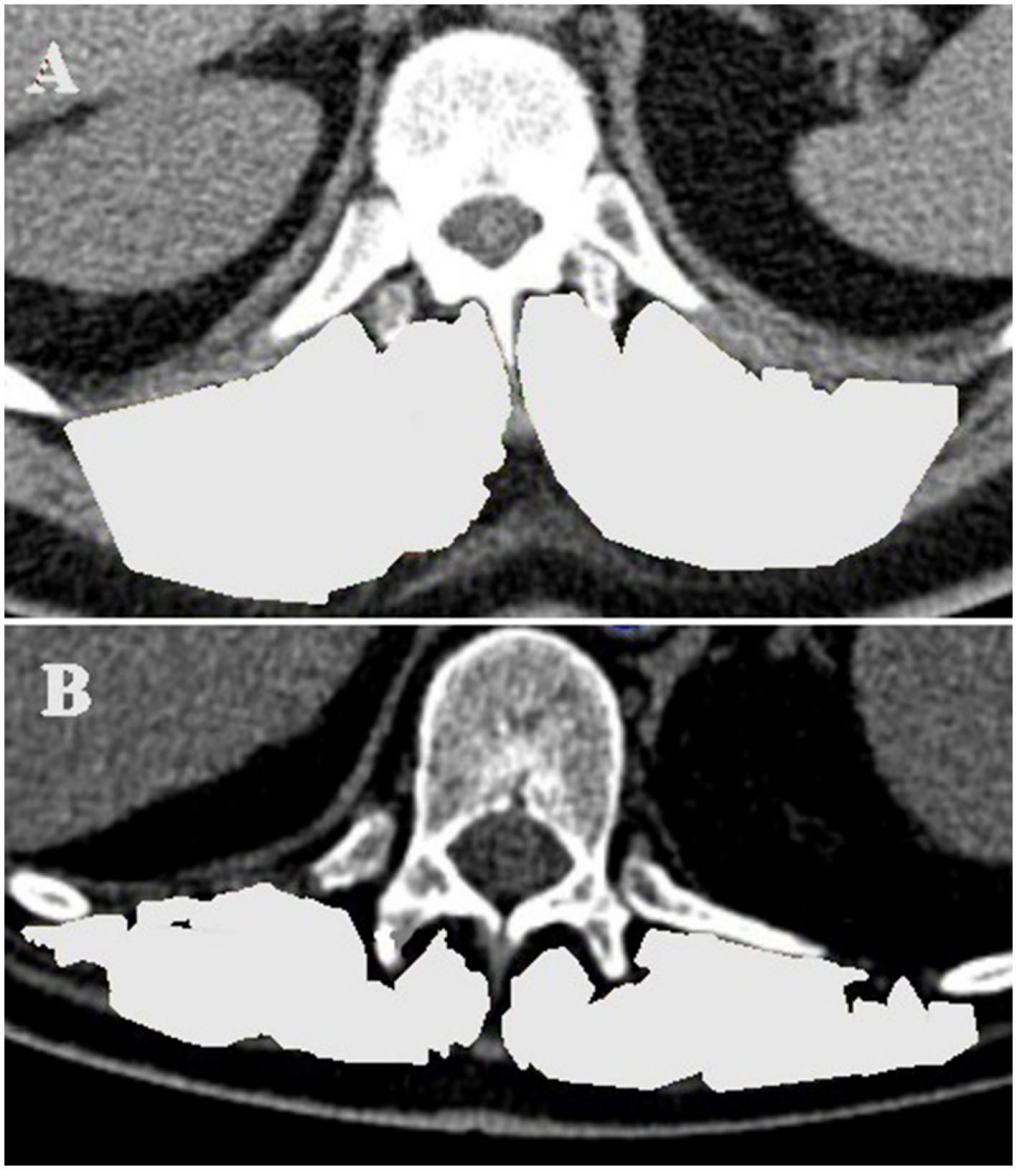
TMESA measurement in an axial CT image at level of the 12th thoracic vertebrae (TMESA area marked in light gray). **(A)** Male patient without impaired muscle area. **(B)** Male patient with impaired muscle area. TMESA, total area of the erector spinae muscles; CT, computed tomography.

To measure muscle mass, the thoracic computed tomography (CT) scan used for the surgical planning and for excluding infection or malignancies during the evaluation process was analyzed with regard to the erector spinae muscles at level Th12 (TMESA) in a single axial image indexed to the body surface area (BSA) estimated with the DuBois formula (15, 16) (Figure 3). Measuring the muscle area in CT scans is considered a gold standard for evaluating muscle mass (6, 17). Wang et al. demonstrated high reliability of image-based estimations of the muscle area (18). The predictive value of the area of the erector spinae muscles was shown in several clinical relations (15, 19, 20). Referring to the results of Minegishi et al., we defined reduced muscle mass as a TMESA/BSA ≤ 17.2 cm^2^/m^2^ (15).

### Statistical Method

Ordinally and nominally scaled parameters are described in absolute (#) and relative (%) terms; between-group comparisons were performed using the χ^2^-test. After testing for a Gaussian distribution, metric values were analyzed using Student's *t*-test or the Mann–Whitney *U*-test, as appropriate. For normally distributed values, the mean value with standard deviation is stated; for other distributions, the median is indicated with the first and third quartile.

Clinically relevant risk factors were examined using univariable logistic regression analysis from which odds ratios (OR) and 95% confidence intervals were calculated. Factors with a *p* < 0.1 in the univariable analysis and gender were included in a multivariable approach, and the best fitting model according to the Akaike Information Criterion was chosen.

Cumulative incidences of death/VAD and transplantation are displayed graphically. Event-free survival during the waiting time (events defined as death and/or emergency VAD implantation) was estimated with a Kaplan–Meier analysis with the time of listing as the starting point. Patients undergoing cardiac transplantation were censored.

All *p*-values should be read descriptively.

## Results

### Baseline Characteristics

**Figure 4:**
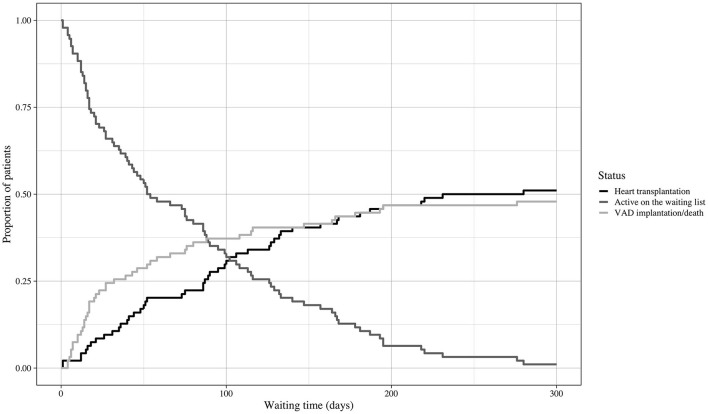
Course of waiting time. Cumulative incidences and course of waiting time. Event: heart transplantation or emergency VAD implantation/death during the HU waiting time. VAD, ventricular assist device; HU, high urgent.

The 49 patients (53%) who underwent transplantation spent 89 days on the HU waiting list; in comparison, patients who did not reach primary transplantation (*n* = 44; 47%) spent 36 days on the waiting list [89 d (IQR 40–130) vs. 35.5 d (IQR 15–93); *p* = 0.007]. The course of the HU waiting time in this cohort is displayed in Figure 4. The groups did not differ in allocation-relevant parameters such as weight, height, gender, age, or blood type. No difference was observed in secondary organ function represented by the glomerular filtration rate (eGFR) calculated with the Modification of Diet in Renal Disease (MDRD) formula and the Model For End-Stage Liver Disease XI (MELD-XI) score (21) (Table 1).

**Table 1:** Baseline characteristics at time of waiting list registration in status “high urgent.”

**Parameter**		**Overall**	**VAD implantation/death**	**Transplantation**	***p*-value**
		**93**	**44**	**49**	
Gender	Male	65 (69.9%)	33 (75.0%)	32 (65.3%)	0.429
	Female	28 (30.1%)	11 (25.0%)	17 (34.7%)	
NYHA	III	14 (15.1%)	4 (9.1%)	10 (20.4%)	0.217
	IV	79 (84.9%)	40 (90.9%)	39 (79.6%)	
INTERMACS	I	1 (1.1%)	0 (0.0%)	1 (2.0%)	0.666
	II	22 (23.7%)	9 (20.5%)	13 (26.5%)	
	III	59 (63.4%)	30 (68.2%)	29 (59.2%)	
	IV–VII	11 (11.8%)	5 (11.4%)	6 (12.2%)	
Age (years)		53 [37, 57]	54 [35, 58]	52 [38, 57]	0.890
Weight (kg)		80.60 [67.10, 90.80]	80.30 [68.70, 91.95]	82.00 [62.00, 90.50]	0.881
Height (cm)		176.00 [170.00, 182.00]	176.50 [171.75, 182.50]	175.00 [168.00, 181.00]	0.349
Survival after surgery (days)		502.00 [152.00, 805.00]	483.50 [144.75, 732.00]	502.00 [156.00, 977.00]	0.618
Time on HU list (days)		54.00 [18.00, 126.00]	29.50 [14.00, 82.00]	89.00 [40.00, 133.00]	0.003
CAS		37.59 [28.20, 52.82]	46.91 [29.46, 62.22]	35.52 [27.17, 43.62]	0.030
Systolic blood pressure (mmHg)		99.00 [91.00, 106.00]	97.00 [90.00, 103.25]	100.00 [92.00, 106.00]	0.183
Diastolic blood pressure (mmHg)		59.00 [55.00, 67.00]	60.50 [56.00, 67.00]	58.00 [55.00, 67.00]	0.709
PAP mean (mmHg)		28.00 [22.00, 33.00]	30.00 [24.00, 38.00]	27.00 [21.00, 31.25]	0.060
PVR (dyn × sec × cm^−5^)		178.50 [133.00, 254.75]	183.00 [139.00, 254.00]	171.00 [123.00, 255.00]	0.584
CVP (mmHg)		10.00 [6.00, 14.00]	8.50 [4.75, 13.25]	10.00 [6.00, 15.00]	0.350
Cardiac index (l/min/m^2^)		2.00 [1.80, 2.30]	2.10 [1.90, 2.40]	1.90 [1.72, 2.10]	0.037
LVEF (%)		20.00 [15.00, 25.00]	19.00 [15.00, 24.25]	20.00 [15.00, 25.00]	0.844
LVEDD (mm)		68.00 [61.00, 76.00]	69.00 [63.50, 78.00]	65.00 [58.00, 74.50]	0.094
Sodium (mmol/L)		137.00 [134.00, 140.00]	136.50 [134.00, 138.25]	137.00 [135.00, 140.00]	0.168
Potassium (mmol/L)		4.30 [4.00, 4.50]	4.35 [4.10, 4.60]	4.20 [3.90, 4.40]	0.023
Hemoglobin (g/dl)		12.00 [10.80, 13.40]	11.30 [10.60, 12.95]	12.60 [11.50, 13.70]	0.018
CRP (mg/dl)		0.94 [0.40, 1.60]	1.30 [0.76, 2.47]	0.73 [0.32, 1.20]	0.001
Creatinine (mg/dl)		1.10 [0.93, 1.40]	1.20 [0.97, 1.50]	1.10 [0.90, 1.40]	0.505
eGFR (ml/min/1.73 m^2^)		63.09 [47.74, 80.96]	64.41 [47.90, 80.81]	62.46 [45.98, 81.72]	0.954
Bilirubin (mg/dl)		1.00 [0.73, 1.50]	1.05 [0.79, 1.72]	0.90 [0.65, 1.40]	0.169
MELD-XI score		11.90 [7.83, 14.88]	13.06 [8.88, 14.87]	10.56 [6.66, 14.88]	0.325
NT-proBNP (pg/ml)		3918.00 [2092.75, 7,343.00]	4282.50 [2756.75, 9200.75]	3265.50 [1821.50, 5688.75]	0.058
Inotropic support	Yes	82 (88.2%)	39 (88.6%)	43 (87.8%)	1.000
	No	11 (11.8%)	5 (11.4%)	6 (12.2%)	
Levosimendan	No	64 (78.0%)	32 (82.1%)	32 (74.4%)	0.571
	Yes	18 (22.0%)	7 (17.9%)	11 (25.6%)	
Dobutamine (dosage)		3.88 [0.00, 5.89]	4.09 [0.00, 7.43]	2.66 [0.00, 5.10]	0.094
Milrinone (dosage)		0.19 [0.00, 0.39]	0.21 [0.00, 0.43]	0.16 [0.00, 0.34]	0.236
Previous cardiac surgery	No	76 (81.7%)	34 (77.3%)	42 (85.7%)	0.434
	Yes	17 (18.3%)	10 (22.7%)	7 (14.3%)	
Cardiac disease	CAD	16 (17.2%)	9 (20.5%)	7 (14.3%)	0.891
	DCMP	56 (60.2%)	27 (61.4%)	29 (59.2%)	
	CHD	7 (7.5%)	3 (6.8%)	4 (8.2%)	
	HCM	2 (2.2%)	1 (2.3%)	1 (2.0%)	
	Other	5 (5.4%)	2 (4.5%)	3 (6.1%)	
	Other CMP	7 (7.5%)	2 (4.5%)	5 (10.2%)	
TMESA/BSA (cm^2^/m^2^)		18.21 [15.93, 22.10]	17.37 [14.87, 20.87]	19.09 [16.14, 22.84]	0.059

*CAS, cardiac allocation score; PAP, pulmonary artery pressure; PVR, pulmonary vascular resistance; CVP, central venous pressure; LVEF, left ventricular ejection fraction; LVEDD, left ventricular end-diastolic volume; CRP, C-reactive protein; eGFR, estimated glomerular filtration rate; CAD, coronary artery disease; DCMP, dilated cardiomyopathy; CHD, congenital heart disease; HCM, hypertrophic cardiomyopathy; CMP, cardiomyopathy*.

*Data are presented as numbers (%), mean (standard deviation), or median [25th quartile, 75th quartile] as appropriate and groups were compared using the Mann–Whitney U test or chi-square test as appropriate*.

**Figure 5:**
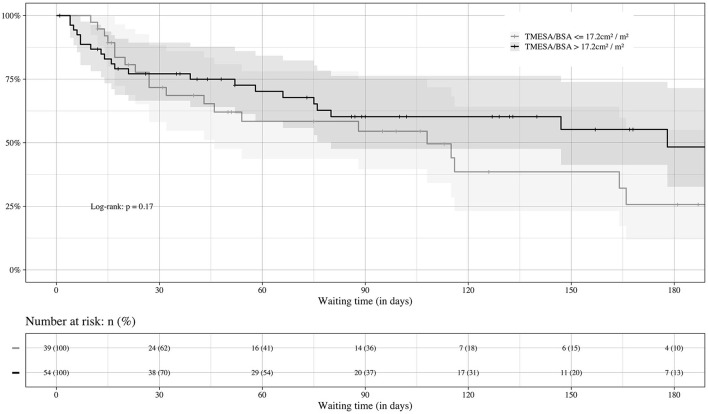
Prognosis of waiting time in regard of sarcopenia. Impact of muscle mass on the course of HU waiting time. Event: death or emergency VAD implantation during the HU waiting time, censored: heart transplantation. VAD, ventricular assist device; TMESA/BSA, total area of the erector spinae muscles/body surface area; HU, high urgent.

Thirty-nine (42%) of the patients had a TMESA/BSA ≤ 17.2 cm^2^/m^2^ [compare Minegishi et al. (15)] and were therefore defined as reduced muscle mass. The impact of muscle mass appeared to increase if the waiting time exceeded 20 days (Figure 5). Patients with a significantly lower TMESA/BSA had a lower creatinine level and MELD-XI score and a higher cardiac index (Table 2).

**Table 2:** Baseline characteristics at time of waiting list registration in status “high urgent”: patients with reduced muscle area vs. patients with TMESA/BSA >17.2 cm^2^/m^2^.

**Parameter**		**Overall**	**TMESA/BSA ≤17.2 cm^**2**^/m^**2**^**	**TMESA/BSA >17.2 cm^**2**^/m^**2**^**	***p*-value**
		**93**	**39**	**54**	
Gender	Male	65 (69.9%)	17 (43.6%)	48 (88.9%)	<0.001
	Female	28 (30.1%)	22 (56.4%)	6 (11.1%)	
NYHA	III	14 (15.1%)	4 (10.3%)	10 (18.5%)	0.420
	IV	79 (84.9%)	35 (89.7%)	44 (81.5%)	
INTERMACS	I	1 (1.1%)	1 (2.6%)	0 (0.0%)	0.866
	II	22 (23.7%)	10 (25.6%)	12 (22.2%)	
	III	59 (63.4%)	23 (59.0%)	36 (66.7%)	
	IV-VII	11 (11.8%)	5 (12.8%)	6 (11.1%)	
Age (years)		53.00 [37, 57]	52.00 [34, 57]	54.00 [44, 57]	0.320
PAP mean (mmHg)		28.00 [22.00, 33.00]	27.00 [22.25, 31.50]	30.00 [22.50, 36.75]	0.177
Cardiac index (L/min/m^2^)		2.00 [1.80, 2.30]	2.10 [1.80, 2.50]	1.90 [1.70, 2.20]	0.035
LVEF (%)		20.00 [15.00, 25.00]	20.00 [15.00, 22.75]	19.50 [15.00, 25.00]	0.412
LVEDD (mm)		6.80 [6.10, 7.60]	6.70 [5.80, 7.55]	6.85 [6.23, 7.68]	0.380
Sodium (mmol/l)		137.00 [134.00, 140.00]	136.00 [133.00, 139.00]	137.00 [135.25, 140.00]	0.036
Potassium (mmol/L)		4.30 [4.00, 4.50]	4.30 [3.90, 4.50]	4.30 [4.03, 4.50]	0.534
Hemoglobin (g/dl)		12.00 [10.80, 13.40]	11.70 [10.65, 13.65]	12.30 [10.93, 13.20]	0.621
CRP (mg/dl)		0.94 [0.40, 1.60]	1.10 [0.39, 1.55]	0.86 [0.43, 1.75]	0.864
Creatinine (mg/dl)		1.10 [0.93, 1.40]	1.00 [0.80, 1.35]	1.25 [0.98, 1.50]	0.022
eGFR (ml/min/1.73 m^2^)		63.09 [47.74, 80.96]	69.47 [47.98, 85.67]	62.43 [46.13, 77.91]	0.346
Bilirubin (mg/dl)		1.00 [0.73, 1.50]	1.10 [0.70, 1.45]	0.97 [0.74, 1.67]	0.791
MELD-XI score		11.90 [7.83, 14.88]	9.57 [6.84, 13.25]	13.23 [8.64, 15.14]	0.038
NT-proBNP (pg/dl)		3918.00 [2092.75, 7343.00]	3329.00 [2529.00, 9389.00]	3967.00 [1829.00, 5871.00]	0.441
Cardiac disease	CAD	16 (17.2%)	5 (12.8%)	11 (20.4%)	0.176
	DCMP	56 (60.2%)	22 (56.4%)	34 (63.0%)	
	GUCH	7 (7.5%)	6 (15.4%)	1 (1.9%)	
	HCM	2 (2.2%)	1 (2.6%)	1 (1.9%)	
	Other	5 (5.4%)	3 (7.7%)	2 (3.7%)	
	Other CMP	7 (7.5%)	2 (5.1%)	5 (9.3%)	

*CAS, cardiac allocation score; PAP, pulmonary artery pressure; LVEF, left ventricular ejection fraction; LVEDD, left ventricular end-diastolic volume; CRP, C-reactive protein; eGFR, estimated glomerular filtration rate; CAD, coronary artery disease; DCMP, dilated cardiomyopathy; CHD, congenital heart disease; HCM, hypertrophic cardiomyopathy; CMP, cardiomyopathy*.

*Data are presented as numbers (%), mean (standard deviation), or median [25th quartile, 75th quartile] as appropriate and groups were compared using the Mann–Whitney U test or chi-square test as appropriate*.

**Figure 6:**
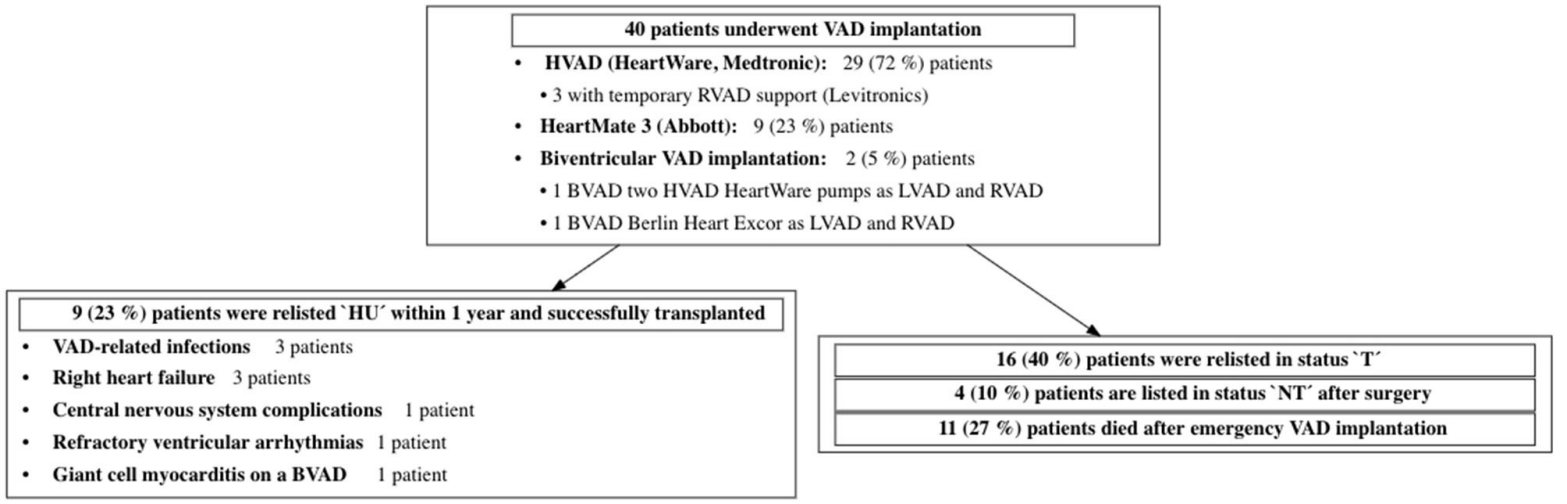
Outcome after emergency VAD implantation during HU waiting time. VAD, ventricular assist device; LVAD, left ventricular assist device; RVAD, right ventricular assist device; BVAD, biventricular assist device; HU, 'high urgent'; T, 'transplantable'; NT, 'not transplantable.

In the vast majority of the patients [*N* = 36 (81%)], VAD implantation was indicated due to cardiogenic shock despite high-dose combined inotropic support after exhausting conservative treatment options; therefore, by definition, these patients were unstable [Interagency Registry for Mechanically Assisted Circulatory Support (INTERMACS) profile 1 or 2] prior to implantation. In 11 (25%) patients, cardiac decompensation was triggered by a systemic infection. The 12-month survival after surgery was 85% (95% CI: 0.75–0.96%) in transplanted patients and 74% (95% CI: 0.61–0.89%) after emergency VAD implantation. The further course of treatment is displayed in Figure 6.

Four patients died during the waiting time: all underwent short-term circulatory support implantation [three extracorporeal membrane oxygenation (ECMO) and one Impella] in INTERMACS profile 1. One patient required mechanical cardiopulmonary resuscitation due to cardiogenic shock; three patients developed combined cardiogenic and septic shock.

### Impact of Muscle Mass

**Figure 7:**
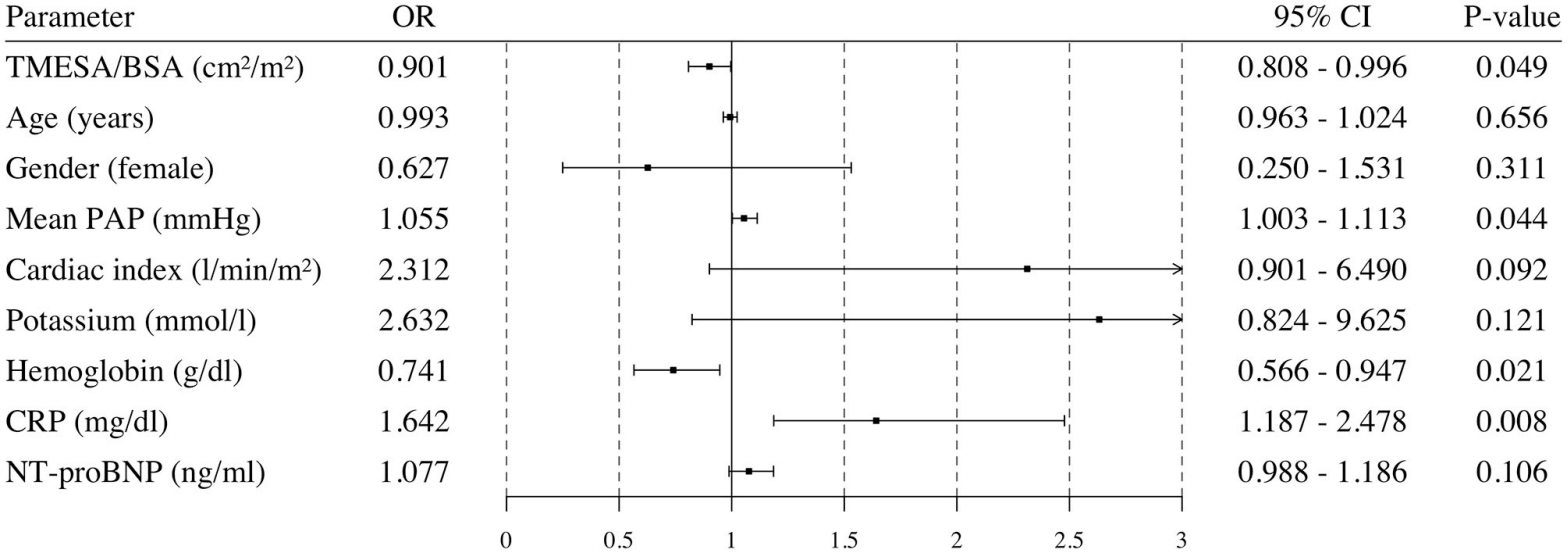
Univariable logistic regression analysis. Odds ratio and 95% confidence interval calculated in a univariable logistic regression analysis for possible risk factors. Endpoint: death or emergency VAD implantation during the HU waiting time. VAD, ventricular assist device; TMESA/BSA, total area of the erector spinae muscles/body surface area; PAP, pulmonary artery pressure; CRP, C-reactive protein; NT-proBNP, N-Terminal Pro-B-Type Natriuretic Peptide; HU, high urgent.

**Figure 8:**
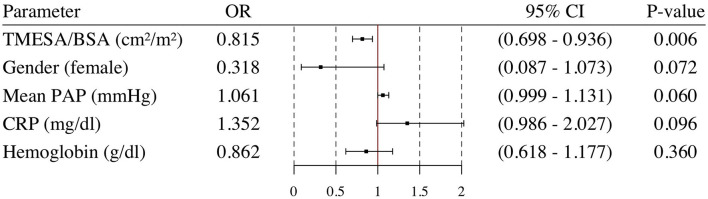
Multivariable logistic regression analysis. Odds ratio and 95% confidence interval calculated in a multivariable logistic regression analysis for TMESA/BSA adjusted for Gender, mean PAP, CRP and hemoglobin. Endpoint: death or emergency VAD implantation during the HU waiting time. VAD, ventricular assist device; TMESA/BSA, total area of the erector spinae muscles/body surface area; PAP, pulmonary artery pressure; CRP, C-reactive protein; HU, high urgent.

Patients who underwent emergency VAD implantation or died during the HU waiting time had a lower TMESA/BSA (Table 1). The univariate logistic analysis revealed a significantly decreased risk of emergency VAD implantation or death during the HU waiting time by 10% for every cm^2^/m^2^ increase in TMESA/BSA [OR 0.901 (95% CI: 0.808–0.996); *p* = 0.049, compare Figure 7]. Further risk factors are displayed in Figure 7. Multivariable analysis adjusted for gender, mean PAP, CRP, and hemoglobin levels revealed muscle mass as an independent risk factor for emergency VAD implantation or death, whereas the other parameters were not significant in this model (Figure 8).

## Discussion

### Impact of Muscle Mass on the Prognosis During the Waiting Time

If the hemodynamic situation deteriorates during the HU waiting time and no suitable donor organ is available, mechanical circulatory support remains the only therapeutic option. Close monitoring for a further decline in cardiac function while waiting for heart transplantation is crucial. If patients met certain criteria, they underwent emergency VAD implantation according to our institutional guidelines (Figure 2).

Muscle mass, represented by TMESA/BSA, was identified as a possible risk factor for emergency VAD implantation or death during the HU waiting time for heart transplantation. It might indicate a reduced resilience against stressors, such as infections or hemodynamic challenges with the need to escalate positive inotropic therapy. Comparable results were published for the impact of frailty, of which sarcopenia can be a symptom, prior to kidney, liver, and lung transplantation, with an increased waiting list mortality for frail and/or sarcopenic patients (7–11). Mostly, a (modified) Fried Frailty Phenotype was used to identify frailty and showed its prognostic value for waiting list mortality irrespective of disease-specific risk factors (5).

Minegishi et al. demonstrated the impact of a reduced TMESA/BSA ≤ 17.2 cm^2^/m^2^ on the outcome after pneumonia (15). Referring to their results, reduced muscle mass was defined by TMESA/BSA ≤ 17.2 cm^2^/m^2^. TMESA/BSA ≤ 17.2 cm^2^/m^2^ was a relevant risk factor for emergency VAD implantation or death during the HU waiting time in advanced heart failure patients. Its impact was independent of gender and other known risk factors such as congestion as an early sign of beginning cardiac deterioration (represented by mean PAP), inflammation (represented by CRP), and anemia (represented by hemoglobin levels). With 42%, the prevalence of reduced muscle mass in our cohort is comparable to the expected prevalence of frailty in this cohort, which is around 45% according to the 2019 position paper on frailty in heart failure patients and the estimated frailty rate of 44.5% in the meta-analysis of Denfeld et al. (22) and Vitale et al. (23).

The impact of reduced muscle mass increases the longer the waiting time is. These findings are in line with our pathophysiological consideration that, in the early phase of waiting, muscle mass and sarcopenia play a less important role compared with deteriorating hemodynamic stability despite inotropic support at the time of listing (4, 24). Our impression is that, unlike in patients awaiting kidney, liver, or lung transplantation, the organ-specific hemodynamic risk of progressing cardiac instability in patients with end-stage heart failure may overlay the potential vulnerability due to reduced muscle mass, especially during the initial period after listing in HU status. Additionally, patients with a reduced muscle mass had a slightly better cardiac index at baseline, giving these patients a possible starting advantage compensating the effect of muscle mass in this early phase. Creatinine levels and MELD-XI scores were lower in the cohort with TMESA/BSA ≤ 17.2 cm^2^/m^2^, possibly owing to lower creatinine levels due to decreased muscle mass.

Adapting the evaluation of muscle mass to the standard monitoring protocol could contribute to identifying patients at risk of decompensation and optimizing the timing of VAD implantation if deterioration occurs.

### CT-Guided Measurement of the Muscle Mass

TMESA/BSA was shown to be an effective method for evaluating muscle mass in these patients, being measurable in the standard thoracic CT scan prior to listing, which is needed in most patients for evaluation, even in emergency situations. In contrast, the iliopsoas muscle, which is widely researched for an evaluation of muscle area (6), would require an additional CT scan of the abdomen, which may not be standard prior to thoracic surgery. Additionally, being a minor muscle, the iliopsoas muscle may not be as representative as other core muscles (6).

Gold standards for diagnosing muscle mass, sarcopenia and frailty in advanced heart failure patients are yet to be defined. One of the most commonly used methods to evaluate frailty is the Fried Frailty Phenotype, which includes with gait speed, grip strength, and weight loss several components of sarcopenia (5). The evaluation of frailty in patients referred for heart transplantation was described by Leng et al. (25) who found an increased post-transplant mortality in frail patients.

However, in patients on inotropic support waiting in the “high urgent” status and/or in UNOS status 3, these assessments lack feasibility, because these patients by definition suffer from severe exercise intolerance or severe ventricular arrhythmias, both of which preclude the estimation of physical performance, making the evaluation of muscle mass even more important. Additionally, results may be skewed by a floor effect due to exercise intolerance caused by heart failure, as described by the Heart Failure Association/European Society of Cardiology (23). This increases the importance of image-based methods for evaluating muscle area that are independent of patients' physical performance, although these methods represent only the muscle mass without considering functional aspect of sarcopenia.

For risk stratification prior to cardiac transplantation, muscle area as a surrogate for biological age could provide valuable information. It should be included in patients' routine evaluation, e.g., to identify patients who would benefit from early VAD implantation or well-defined prehabilitation programs, as will be assessed for instance in the upcoming trial of Stoppe et al., who are planning an interventional trial with a predefined nutritional and physical exercise program prior to VAD implantation in stable heart failure patients (26).

### Further Implications of Including Muscle Mass Measurement in Patients' Evaluation

As sarcopenia is potentially reversible, both muscle mass and function could be positively influenced by following a healthy diet and starting a clinically adapted exercise program, or at least by engaging in passive movement support under close monitoring to prevent a further decline in physical reserve. In their review, McCann et al. identified several influenceable domains and showed the impact of preoperative rehabilitation prior to cardiac surgery on the improvement of the postoperative outcome (27). Developing a suitable intervention program for advanced heart failure patients could be the next step in optimizing the outcome while on the waiting list and after surgery, as suggested by Leng and Kittleson (25). Gimeno-Santos et al. were able to demonstrate the feasibility, safety, and potential benefit of a tailored exercise program during the waiting time for heart transplantation in 19 patients (28).

In some patients, VAD implantation could be a therapeutic option for disease-dependent sarcopenia, thereby optimizing the patient's status prior to cardiac transplantation. Maurer et al. and Jha et al. demonstrated the potential reversibility of frailty and therefore of impaired muscle function 2–6 months after VAD implantation without an exercise program when heart failure treatment was sufficient (29, 30). Physical training in particular should be safer and more efficient in VAD patients than in patients on inotropic support waiting without a VAD. The position paper of the European Society of Cardiology by Adamopoulos et al. highlights the benefits and limitations of exercise training in VAD patients (31). Although the surgery is more complex in patients on VAD support prior to heart transplantation, the outcome of patients with and without VAD implantation prior to cardiac transplantation is comparable (32).

### Possible Strategy Based on the Findings

Muscle area evaluation seems to be a valuable tool for additional risk stratification. The standard evaluation process of advanced heart failure patients should include screening for reduced muscle mass.

Based on gender, weight, height, blood type, and availability of donor organs, the waiting time can be estimated and differs between regions. In patients with a reduced muscle mass (TMESA/BSA ≤ 17.2 cm^2^/m^2^) and longer estimated waiting times, VAD implantation as a “bridge to transplantability” and specialized prehabilitation and nutrition programs to stop the progression of heart failure-associated cachexia, sarcopenia, and frailty should be considered as a possible treatment approach.

### Study Limitations

This cohort represents the experience of a single center; therefore, it is not robust against systematic influencers caused by intrahospital standard procedures, such as the timing of emergency VAD implantation. In all our patients, long-term mechanical circulatory support with loss of the status “high urgent” was chosen, where possible, over stabilization with a short-term circulatory support like ECMO or Impella and continued listing in the high-urgency status, since the length of the waiting time is not predictable.

Patients were spared additional radiation in this retrospective analysis by using the standard CT for the evaluation; however, a more standardized protocol including the exact timing of the CT and standardization of the contrast agent regimen might improve the significance and allow the measurement of muscle density as a potential surrogate for muscle quality.

According to our analysis, the impact of reduced muscle mass increases with the waiting time. Although this can be explained pathophysiologically, it warrants the support of prospective studies.

## Conclusions

Muscle mass represented by TMESA/BSA at the time of listing for heart transplantation was a relevant risk factor for emergency VAD implantation or death during the HU waiting time prior to heart transplantation. Routine evaluation of muscle mass could identify patients at risk for decompensation during the waiting time and could help optimize the timing of VAD support.

## Data Availability Statement

The data analyzed in this study is subject to the following licenses/restrictions: Privacy policy for medical data. Requests to access these datasets should be directed to roehrich@dhzb.de.

## Ethics Statement

The studies involving human participants were reviewed and approved by Ethics Committee of Charité–Universitätsmedizin Berlin (EA2/236/17). Written informed consent to participate in this study was provided by the participants' legal guardian/next of kin.

## Author Contributions

LR and FS administrative support and collection and assembly of data. LR, IJ, LK, NS, MH, JK, EP, CK, VF, and FS provision of study materials or patients. LR, SS, SK, JSt, and FS statistics, data analysis, and interpretation. All the authors concept and design, article draft, critical revision of article, and approval of article.

## Funding

LR reports a research grant (Kaltenbach doctoral stipendium) from German Heart Foundation during the conduct of the study (K/38/18).

## Conflict of Interest

VF reports grants from Medtronic GmbH, Abbottt GmbH & Co. KG, Boston Scientific, Edwards Lifesciences, JOTEC/CryoLife and other financial activities from Berlin Heart, Biotronik SE & Co., Novartis Pharma GmbH, Zurich Heart outside of the submitted work. FS reports other financial activities from Novartis, Abbott, Orion Pharma, Astra Zeneca and non-financial support from Medtronic outside of the submitted work. LR reports grants from German Heart Foundation during the conduct of the study, share holdings of Alianz SE, Carl Zeiss Meditec AG, CompuGroup Medical SE & Co. KGaA, Evotec SE, Fresenius Medical Care AG & Co. KGaA outside of the submitted work. The remaining authors declare that the research was conducted in the absence of any commercial or financial relationships that could be construed as a potential conflict of interest.

## Publisher's Note

All claims expressed in this article are solely those of the authors and do not necessarily represent those of their affiliated organizations, or those of the publisher, the editors and the reviewers. Any product that may be evaluated in this article, or claim that may be made by its manufacturer, is not guaranteed or endorsed by the publisher.
